# Real‐world outcomes among patients with EGFR‐mutated non‐small cell lung cancer treated with EGFR tyrosine kinase inhibitors versus immunotherapy or chemotherapy in the first‐line setting

**DOI:** 10.1002/cam4.6052

**Published:** 2023-06-12

**Authors:** Jon Apple, Rahul Shenolikar, Kevin De Silva, Ping Sun, Alexander Spira

**Affiliations:** ^1^ AstraZeneca Pharmaceuticals LP Gaithersburg Maryland USA; ^2^ Virginia Cancer Specialists Fairfax Virginia USA

**Keywords:** biomarkers, chemotherapy, lung cancer, target therapy, tyrosine kinase inhibitors

## Abstract

**Background:**

Despite national guideline recommendations, epidermal growth factor receptor mutated (EGFRm) metastatic non‐small cell lung cancer (mNSCLC) patients may still receive suboptimal treatment in the first line (1L). This study evaluated 1L therapy initiation in relation to biomarker testing results and time to next‐treatment or death (TTNTD) in patients receiving EGFR tyrosine kinase inhibitors (TKIs) versus immunotherapy (IO) or chemotherapy.

**Methods:**

Stage IV EGFRm mNSCLC adults that initiated 1L EGFR TKI (first, second, or third generation), IO ± chemotherapy (IO users), or chemotherapy alone from 5/2017–12/2019 were identified from the Flatiron database. Logistic regression estimated the likelihood of initiating treatment before receiving testing results for each therapy. Median TTNTD was evaluated via Kaplan–Meier analysis. Adjusted hazards ratios (HRs) and 95% CI examining the association of 1L therapy with TTNTD were reported from multivariable Cox proportional‐hazards models.

**Results:**

Among 758 EGFRm mNSCLC patients, EGFR TKI was used as 1L therapy for 87.3% of patients (*n* = 662), IO in 8.3% (*n* = 63), and chemotherapy only in 4.4% (*n* = 33). The majority of IO (61.9%) and chemotherapy only patients (60.6%) initiated therapy before test results were available, compared to 9.7% of EGFR TKIs. The odds of initiating therapy before receiving test results were higher for IO (OR: 19.6, *p* < 0.001) and chemotherapy alone (OR: 14.1, *p* < 0.001) in comparison to EGFR TKIs. Compared to IO and chemotherapy, EGFR TKIs had longer median TTNTD (EGFR TKI: 14.8 months, 95% CI: 13.5–16.3; IO: 3.7 months, 95% CI 2.8–6.2; chemotherapy: 4.4 months, 95% CI 3.1–6.8, *p* < 0.001). EGFR TKI patients had significantly lower risk of initiating second‐line therapy or death compared to patients on 1L IO (HR: 0.33, *p* < 0.001) or 1L chemotherapy (HR: 0.34, *p* < 0.001).

**Conclusions:**

A portion of biomarker testing results were not used to guide 1L therapy. Patients initiating EGFR TKI as 1L therapy had longer TTNTD than IO or chemotherapy.

## 1 INTRODUCTION

Lung cancer is the most common cause of cancer‐related death in the United States with an estimated 130,000 deaths in 2022 alone.^1^ Non‐small cell lung cancer (NSCLC) contributes to approximately 85% of these cases every year.^2^ Nearly 55% of lung cancer cases are diagnosed with metastatic, stage IV disease which has a 5‐year survival rate of only 6%.^3^ Advances in molecular genomics have led to the identification of driver mutations such as epidermal growth factor receptor (EGFR) and anaplastic lymphoma kinase (ALK).^4^ In western populations, EGFR mutations (EGFRm) can be found in approximately 19% of patients with adenocarcinoma NSCLC subtypes.^5^ Several randomized phase III trials in stage IV NSCLC patients have shown better tumor response and progression‐free survival (PFS) for EGFR tyrosine kinase inhibitors (TKIs) compared to platinum‐based doublet chemotherapy.^6^, ^7^, ^8^ EGFR TKIs are now the NCCN Clinical Practice Guidelines in Oncology (NCCN Guidelines®) recommended first‐line (1L) treatment.^9^

More recently, osimertinib, a third‐generation EGFR TKI,^9^ demonstrated superiority over first‐generation (erlotinib or gefitinib) EGFR TKIs. Specifically, in the Phase III FLAURA trial, patients treated with 1L osimertinib demonstrated prolonged PFS (18.9 vs. 10.2 months) and overall survival (OS) (38.6 vs. 31.8 months) compared to erlotinib or gefitinib.^10^, ^11^ This led to the approval of osimertinib in April 2018, which has now become the frontline standard of care for patients with stage IV EGFRm NSCLC in the United States.

While EGFR TKIs are the NCCN Guidelines® recommended 1L therapy for patients harboring EGFRm such as exon 19 deletions or L858R mutations, almost 20% of EGFRm NSCLC patients are prescribed immunotherapy (IO) with or without chemotherapy or chemotherapy alone.^12^ Patients require biomarker testing to be identified as having a targetable EGFR mutation in order to receive an EGFR TKI. One of the biggest challenges to biomarker testing is the turnaround time (TAT) which can sometimes exceed 2 weeks.^13^ Given the long TAT for biomarker testing and the aggressiveness of stage IV NSCLC,^14^ prescribers may initiate IO with or without chemotherapy before testing results are received. While starting treatment earlier may seem like the best approach, inappropriate therapies in these patients could result in suboptimal outcomes. Results from a study conducted by the US Food and Drug Administration showed that patients with targetable mutations, such as EGFR or ALK, that received IO at any point in therapy had lower OS compared to patients without targetable mutations (4.7 vs. 8.6 months, respectively).^15^ Additionally, even in EGFRm patients with high programmed death ligand 1 (PD‐L1) expression, which correlates to better outcomes with IO therapies in patients without mutations,^16^ response rates are very limited.^17^ Findings from these studies highlight the importance of adhering to guideline‐recommended treatments in patients with targetable mutations.

While the benefit of EGFR TKIs over chemotherapy has been demonstrated in several clinical studies, data on real‐world outcomes comparing 1L EGFR TKI treatment to IO treatment in patients with stage IV EGFRm NSCLC is limited. Two recent cohort studies found that patients with stage IIIB/IV EGFRm NSCLC treated with EGFR TKIs had significantly longer time‐to‐next treatment or death (TTNTD), when compared to those treated with other 1L therapies such as chemotherapy.^12^, ^18^ However, both studies included a broader patient population (other histologies, locally advanced stage, recurrent disease) and did not perform direct comparisons to IO. Additionally, these studies utilized older data and did not include or report the inclusion of osimertinib, the current standard of care, in their assessment. To address these gaps in the literature, this study evaluated the 1L initiation in relation to biomarker testing results and TTNTD among patients with stage IV EGFRm NSCLC treated with 1L EGFR TKI (i.e., osimertinib, erlotinib, afatinib, and gefitinib), IO with or without chemotherapy, or chemotherapy alone.

## 2 METHODS

### 2.1 Data source

This study used data from the Flatiron Health Electronic Health Record (EHR)‐derived database, which contains longitudinal, patient‐level data from a demographically and geographically diverse, nationally representative population in the United States.^19^ At the time of analysis, the database included data from around 280 community practices and academic institutions, representing approximately 800 distinct sites of patient care and more than 2.2 million active patients with cancer in the United States. Of these patients, over 65,000 were diagnosed with stage IV NSCLC. Data were updated monthly and contained structured and unstructured information abstracted from medical notes, as well as other unstructured documents such as radiology, pathology, biomarker reports, and patient discharge summaries. The data are de‐identified and provisions were in place to prevent re‐identification to protect patient confidentiality. This is in accordance with the 1996 Health Information Portability and Accountability Act.

### 2.2 Study design

A retrospective longitudinal cohort study was conducted among adult patients with stage IV EGFRm NSCLC who were treated with EGFR TKI, IO with or without chemotherapy (defined as IO users in this study), or chemotherapy alone. Patients were included if they had a diagnosis for lung cancer (International Classification of Diseases [ICD], 9th Revision, Clinical Modification [CM] codes: 162.x; ICD, 10th Revision, CM codes: C34.x and C39.9), ≥2 documented clinical visits on or after January 1, 2011, pathology consistent with NSCLC, and a diagnosis with stage IVA or IVB NSCLC. Additionally, patients were required to be at least 18 years of age at the time of their diagnosis and have tested positive for at least one common EGFR mutation (exon 19 deletion or exon 21 L858R point mutation) during the study period. The index date was defined as the date of initiation of 1L NSCLC therapy, which must have been initiated within 1 year following a patient's stage IV NSCLC diagnosis. In addition, 1L NSCLC therapy must have been initiated on or after pembrolizumab and chemotherapy was approved for advanced NSCLC on May 10, 2017 and before December 31, 2019, to allow for ≥6 months of follow‐up. Patients were excluded if they had a history of any cancer prior to the index date, had evidence of squamous cell etiology, or if they received a 1L clinical study drug therapy. Furthermore, patients who received subsequent therapy within 8 weeks of receiving biomarker test results were excluded as to avoid misclassifying them as those who progressed as they may have switched to a different therapy due to receipt of biomarker test results rather than actual disease progression.

Outcomes were evaluated during the observation (follow‐up) period, which spanned from the index date to the earliest of death or end of data availability (June 30, 2020). The baseline period was defined as the 3‐month period prior to the index date, during which demographic and clinical characteristics were evaluated.

### 2.3 Study measures

Demographic and clinical variables assessed included age, gender, race, ethnicity, geographic region, year of 1L NSCLC therapy initiation, insurance type, smoking history, physician practice type (academic or community centers), National Cancer Institute (NCI)‐Charlson comorbidities, NSCLC characteristics (i.e., cancer stage at diagnosis and time from diagnosis to index date), Eastern Cooperative Oncology Group (ECOG) performance status, and EGFR mutation information. ECOG scores closest to and within 3 months of the index date were used. For patients with multiple ECOG scores meeting the above criteria, the highest ECOG score was used.

1L NSCLC therapy was identified based on Flatiron Health's pre‐defined algorithm for line of therapy, which has been described in a prior publication.^20^ In brief, the start of 1L therapy was defined as the first episode of an eligible therapy that was given after or up to 14 days before the patient's date of diagnosis with stage IV NSCLC. Combination therapy was considered as other eligible NSCLC therapy given within 28 days of the first eligible therapy. When a treatment gap of more than 120 days occurred, the line of therapy was advanced.

Odds of therapy initiation before biomarker testing results and TTNTD were the outcomes of interest in this study. 1L initiation before biomarker testing result is defined as a 1L therapy initiation date that precedes an EGFR testing result date. TTNTD serves as a proxy for PFS. TTNTD was defined as the time from initiation of 1L NSCLC therapy to the day prior to initiation of second‐line (2L) therapy or death. Patients with no evidence of 2L or death were censored at the end of data availability. Dates of death were determined based on data combined from two data sources, including the Social Security Death Index and a commercial mortality dataset, and linked to patient‐level data in the Flatiron database. Month level data were available; however, the exact date of death was unavailable due to de‐identification processing, therefore, the day of death was imputed as the 15th of the month.

### 2.4 Statistical analysis

Baseline demographic and clinical characteristics were described overall and stratified by type of 1L NSCLC therapy. In addition, treatment patterns were characterized with respect to timing of EGFRm test results. Means, standard deviations, and medians were reported for continuous variables, whereas frequencies and percentages were reported for categorical variables. Comparisons between EGFR TKI and IO or chemotherapy alone were made using Wilcoxon rank sum tests for continuous variables and chi‐square or Fisher's exact tests for categorical variables.

Logistic regression, controlling for age, sex, race, region, history of smoking, year of 1L initiation, histology, NCI index, time from stage IV NSCLC diagnosis to index date, and ECOG score, was used to estimate the odds ratio and 95% Wald CI to understand the association between type of 1L therapy prescribed and timing of availability of EGFR testing results. Kaplan–Meier (KM) analysis was conducted to estimate median TTNTD and respective 95% confidence intervals (CIs) by 1L therapy. Log‐rank tests were used to compare KM curves between patients treated with 1L EGFR TKIs versus IO or chemotherapy, separately. Multivariable Cox proportional‐hazards models, controlling for the same variables above, were used to estimate hazard ratios (HRs) and corresponding 95% CIs for the effect of EGFR TKI versus IO or chemotherapy, separately, on TTNTD. All analyses were conducted in SAS 9.4 (SAS Institute).

## 3 RESULTS

### 3.1 Patient demographic and clinical characteristics

**FIGURE 1:**
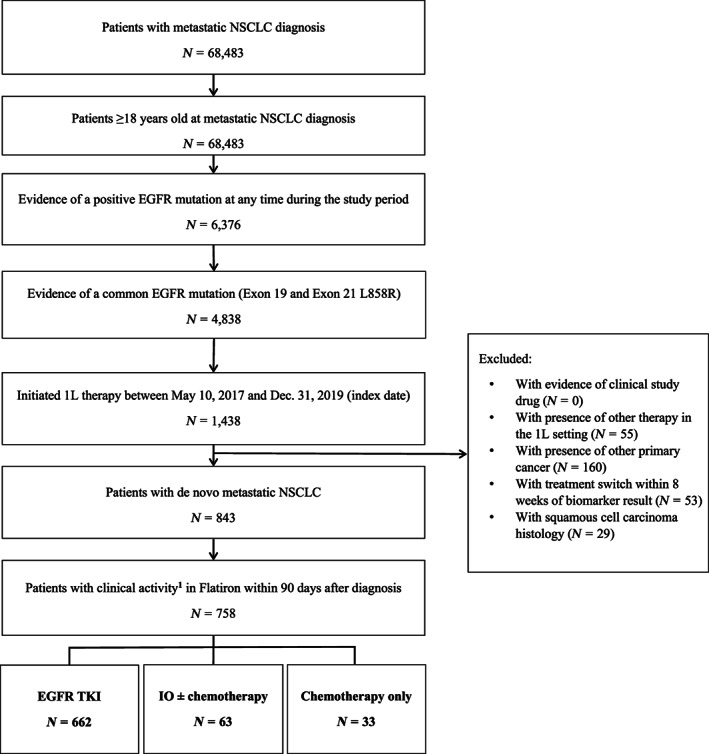
Patient attrition. 1L, first line; EGFR, epidermal growth factor receptor; EGFRm, EGFR mutation; IO, immunotherapy; NSCLC, non‐small cell lung cancer; TKI, tyrosine kinase inhibitor. ^1^Clinical activity included visits, start of any line of therapy, lab tests, vital assessments, ECOG assessments, or comorbidity diagnoses.

A total of 68,483 patients with stage IV mNSCLC were identified in the Flatiron database from May 2017 to December 2019. Of these patients, a final analytical sample of 758 EGFRm patients was identified after applying the inclusion/exclusion criteria (Figure 1).

Of the 758 patients, 662 (87.3%) received 1L treatment with EGFR TKI, 62 (8.3%) received IO (monotherapy or in combination with chemotherapy), and 33 (4.4%) received chemotherapy alone (Table 1). The mean age was 67.6 years and was similar across all three treatment groups. Patients who received 1L EGFR TKI were significantly more likely to be female (67.1% vs. 48.5.0%, *p* = 0.028), initiated therapy in 2019 (40.9% vs. 21.2%, *p* = 0.024), and had a history of smoking (42.7% vs. 52.5%, *p* = 0.002), compared to patients who received chemotherapy alone. Most patients on EGFR TKIs and all patients on chemotherapy were treated in the community setting (87.5% vs. 100%, *p* = 0.030). Differences in practice setting and smoking history between patients treated with EGFR TKI and IO were not statistically significant. For patients who received EGFR TKI, the median time from stage IV NSCLC diagnosis to 1L NSCLC therapy initiation (i.e., index date) was significantly longer than that among patients who initiated chemotherapy (35.0 vs. 27.0 days, *p* = 0.018) but not for IO therapy (35.0 vs. 30.0 days, *p* = 0.09). Among patients receiving EGFR TKI, exon 19 deletions were similar compared to IO (EGFR TKI: 56.5% vs. IO: 54.0%, *p* = 0.699) and chemotherapy (45.5%, *p* = 0.212).

**TABLE 1:** Baseline demographic and clinical characteristics^a^.

Characteristics	All patients	Patients treated with 1L EGFR TKI	Patients treated with 1L IO	*p* value^b^	Patients treated with 1L chemotherapy only	*p* value^c^
	*N* = 758	*N* = 662	*N* = 63		*N* = 33	
*Demographics*						
Age at index date, years, mean ± SD [median]^d^	67.6 ± 10.9 [69.0]	67.8 ± 11.0 [69.0]	66.4 ± 9.3 [66.0]	0.149	66.8 ± 11.0 [70.0]	0.550
Female, *n* (%)	497 (65.6)	444 (67.1)	37 (58.7)	0.181	16 (48.5)	0.028*
Race, *n* (%)						
White	393 (51.8)	344 (52.0)	34 (54.0)	0.761	15 (45.5)	0.465
Asian	112 (14.8)	103 (15.6)	7 (11.1)	0.347	2 (6.1)	0.137
Black or African American	50 (6.6)	42 (6.3)	6 (9.5)	0.332	2 (6.1)	0.948
Hispanic	2 (0.3)	2 (0.3)	0 (0.0)	0.662	0 (0.0)	0.752
Other	100 (13.2)	87 (13.1)	7 (11.1)	0.647	6 (18.2)	0.407
Unknown	101 (13.3)	84 (12.7)	9 (14.3)	0.717	8 (24.2)	0.056
Geographic region, *n* (%)						
Northeast	138 (18.2)	119 (18.0)	14 (22.2)	0.405	5 (15.2)	0.304
West	181 (23.9)	157 (23.7)	17 (27.6)	0.562	7 (21.2)	0.504
Midwest	82 (10.8)	73 (11.0)	7 (11.1)	0.984	2 (6.1)	0.753
South	256 (33.8)	219 (33.1)	19 (30.2)	0.637	18 (54.5)	0.011*
Unknown	101 (13.3)	94 (14.2)	6 (9.5)	0.304	1 (3.0)	0.231
Physician practice type, *n* (%)						
Academic	86 (11.3)	83 (12.5)	3 (4.8)	0.068	0 (0.0)	0.030*
Community centers	672 (88.7)	579 (87.5)	60 (95.2)	0.068	33 (100.0)	0.030*
Year of initiation of 1L therapy						
2017	176 (23.2)	156 (23.6)	9 (14.3)	0.093	11 (33.3)	0.199
2018	279 (36.8)	235 (35.5)	29 (46.0)	0.097	15 (45.5)	0.245
2019	303 (40.0)	271 (40.9)	25 (39.7)	0.847	7 (21.2)	0.024*
Insurance type, *n* (%)^e^ ^,^ ^f^						
Commercial health plan	317 (41.8)	278 (42.0)	27 (42.9)	0.895	12 (36.4)	0.783
Medicare	175 (23.1)	151 (22.8)	16 (25.4)	0.641	8 (24.2)	0.644
Other payer	189 (24.9)	168 (25.4)	13 (20.6)	0.406	8 (24.2)	0.529
Patient assistance program	22 (2.9)	17 (2.6)	2 (3.2)	0.773	3 (9.1)	0.038*
Medicaid	21 (2.8)	20 (3.0)	0 (0.0)	0.162	1 (3.0)	0.615
Other government program	14 (1.8)	11 (1.7)	3 (4.8)	0.088	0 (0.0)	0.008*
Self‐pay	8 (1.1)	6 (0.9)	1 (1.6)	0.597	1 (3.0)	0.043*
Unknown	12 (1.6)	11 (1.7)	1 (1.6)	0.965	0 (0.0)	0.335
*Clinical characteristics*						
Smoking status, *n* (%)^g^						
History of smoking	336 (44.3)	283 (42.7)	30 (47.6)	0.456	23 (69.7)	0.002*
No history of smoking	422 (55.7)	379 (57.3)	33 (52.4)	0.456	10 (30.3)	0.002*
NCI‐Charlson comorbidities, mean ± SD [median]^h^ ^,^ ^i^	0.2 ± 0.51 [0.0]	0.2 ± 0.52 [0.0]	0.1 ± 0.43 [0.0]	0.927	0.1 ± 0.29 [0.0]	0.734
*NSCLC characteristics*						
Time from stage IV NSCLC diagnosis to index date, days						
Mean ± SD	41.9 ± 44.29	42.7 ± 46.3	38.8 ± 29.3	0.089	32.6 ± 19.5	0.018*
Median (range)	35.0 (10.0, 890.0)	45.0 (1.0, 890.0)	30.0 (8.0, 159.0)	0.089	27.0 (8.0, 84.0)	0.018*
Histology, *n* (%)^j^						
Non‐squamous cell carcinoma	746 (98.4)	651 (98.3)	62 (98.4)	0.965	33 (100)	0.455
NSCLC histology‐NOS	12 (1.6)	11 (1.7)	1 (1.6)	0.965	0 (0.0)	0.455
ECOG grade, *n* (%)^k^						
0	213 (28.1)	179 (27.0)	22 (34.9)	0.182	12 (36.4)	0.242
1	228 (30.1)	197 (29.8)	22 (34.9)	0.394	9 (27.3)	0.760
2	86 (11.3)	79 (11.9)	4 (6.3)	0.183	3 (9.1)	0.621
3	24 (3.2)	23 (3.5)	0 (0.0)	0.133	1 (3.0)	0.892
4	2 (0.3)	1 (0.2)	1 (1.6)	0.038*	0 (0.0)	0.823
Unknown	205 (27.0)	183 (27.6)	14 (22.2)	0.355	8 (24.2)	0.669
EGFR mutation type, *n* (%)^l^						
Exon 19 deletion	423 (55.8)	374 (56.5)	34 (54.0)	0.699	15 (45.5)	0.212
L858R point mutation in exon 21	335 (44.2)	288 (43.5)	29 (46.0)	0.699	18 (54.5)	0.212

Abbreviations: 1L, first‐line therapy; ECOG, Eastern Cooperative Oncology Group; EGFR, epidermal growth factor receptor; IO, immunotherapy; MI, myocardial infarction; NCI, National Cancer Institute; NGS, next‐generation sequencing; NOS = not otherwise specified; NSCLC, non‐small cell lung cancer; SD, standard deviation; TKI, tyrosine kinase inhibitor.

^a^ Unless indicated otherwise, baseline characteristics were evaluated during the three‐month period before the start of first‐line (1L) therapy (index date).

^b^ *p* value compares patients treated with 1L EGFR TKI versus 1L IO.

^c^ *p* value compares patients treated with 1L EGFR TKI versus 1L chemotherapy alone.

^d^ Age was defined at initiation of 1L therapy (index date).

^e^ Insurance was included if the start or end of coverage was within 3 months of the index date.

^f^ Patients may have primary and secondary insurance (i.e., patients may have more than one type of insurance). Unknown insurance type was defined as patients without any of the specified types of insurance (i.e., commercial health plan, Medicaid, Medicare, other government program, other payer, patient assistance program, or self pay).

^g^ Start date of smoking status was not reported in the Flatiron Health Oncoanalytics Database.

^h^ The NCI comorbidity index calculation from the NCI Comorbidity Technical Report by Stedman et al. (2019)^24^ was used.

^i^ Acute MI was not included as an NCI comorbidity as it requires information on the length of inpatient hospital stay, which is unavailable in Flatiron Health Oncoanalytics Database.

^j^ Evaluated at the initial diagnosis of NSCLC.

^k^ ECOG scores closest to and within 3 months of the index date were included. If the patient had multiple ECOG scores, either on the same day closest to the index date, or on multiple days equidistant from the index date, the maximum ECOG score was used.

^l^ Includes all positive EGFR results, regardless of the timing of the test. Patients may have multiple positive EGFR tests, and thus more than one sample type, test type, and mutation type recorded.

* *p* value <0.05.

### 3.2 1L initiation in relation to EGFR testing result

A larger proportion of patients receiving 1L IO (61.9%) or 1L chemotherapy (60.6%) initiated therapy before receiving EGFR testing results compared to patients starting 1L EGFR TKIs (9.4%) (Table 2). Logistic regression showed that compared to EGFR TKIs, the odds of initiating 1L treatment before receiving EGFR test results were much higher for IO (OR: 19.6, 95% CI: 10.4–37.1, *p* < 0.001) and chemotherapy (OR: 14.1, 95% CI: 6.3–31.9, *p* < 0.001) users, respectively (Table 3).

**TABLE 2:** Type of therapy received by timing of 1L initiation before versus after receipt of positive EGFR mutation test results.

		Patients who tested positive for EGFR mutation^a^	
		*N* = 756	
	1L therapy	1L initiation before EGFR testing results available	1L initiation after EGFR testing results available
	*N* = 756	*N* = 121	*N* = 635
Therapy type^b^			
EGFR TKI, *n* (%)	660 (87.3)	62 (9.4)	598 (90.6)
IO, *n* (%)	63 (8.3)	39 (61.9)	24 (38.1)
Chemotherapy only, *n* (%)	33 (4.4)	20 (60.6)	13 (39.4)

Abbreviations: 1L, first line; EGFR: epidermal growth factor receptor; EGFRm: EGFR mutation; IO: immun‐oncology; NSCLC: non‐small cell lung cancer; TKI: tyrosine kinase inhibitor.

^a^ The date of the first positive EGFR test result was used. Patients were excluded if they received their EGFRm test result after 2L therapy initiation (two patients in the EGFR TKI group).

^b^ Categories for drugs were mutually exclusive and were defined as follows:

1. EGFR TKI: treatment with afatinib, erlotinib, gefitinib, or osimertinib.

2. IO: no treatment with EGFR TKI, and treatment with atezolizumab, durvalumab, ipilimumab, nivolumab, or pembrolizumab ± chemotherapy.

3. Chemotherapy only: no treatment with EGFR TKI, IO or any combination therapy, and treatment with carboplatin, cisplatin, docetaxel, etoposide, gemcitabine, paclitaxel, pemetrexed, or vinorelbine.

**TABLE 3:** Odds of initiating 1L therapy before receiving EGFR testing results.

Characteristic	OR (95% CI)
Treatment class	
IO	19.63 (10.39–37.06)*
Chemotherapy only	14.14 (6.27–31.89)*
EGFR TKI (reference)	–
Sex	
Female	0.745 (0.466–1.19)
Male (reference)	–
Race	
Asian	0.95 (0.46–1.96)
Black or African American	0.70 (0.25–1.95)
Other	0.61 (0.27–1.38)
Unknown	1.21 (0.49–3.03)
Caucasian (reference)	–
Region	
Northeast	1.01 (0.42–2.41)
South	0.96 (0.43–2.17)
West	0.79 (0.32–1.94)
Unknown	1.21 (0.49–3.03)
Midwest (reference)	–
Smoking status	
History of smoking	1.16 (0.72–1.86)
No history of smoking (reference)	–
Histology	
Non‐squamous cell carcinoma	0.51 (0.10–2.58)
NOS (reference)	–
ECOG category	
2, 3, 4	1.31 (0.82–2.12)
0, 1 (reference)	–
CCI category	–
≥1	1.43 (0.73–2.82)
0 (reference)	–
Index year	0.75 (0.56–1.02)
Time from diagnosis to index^a^	0.97 (0.96–0.99)*
Age at mNSCLC diagnosis	0.99 (0.97–1.02)

Abbreviations: 1L: first line; CCI: Charlson Comorbidity Index; CI: confidence interval; ECOG: Eastern Cooperative Oncology Group; EGFR: epidermal growth factor receptor; IO: immunotherapy; mNSCLC: metastatic non‐small cell lung cancer; NOS: not otherwise specified; OR: odds ratio; TKI: tyrosine kinase inhibitor.

^a^ Index is the initiation of 1L mNSCLC therapy.

* *p* value <0.05.

### 3.3 Time to next‐treatment or death

**FIGURE 2:**
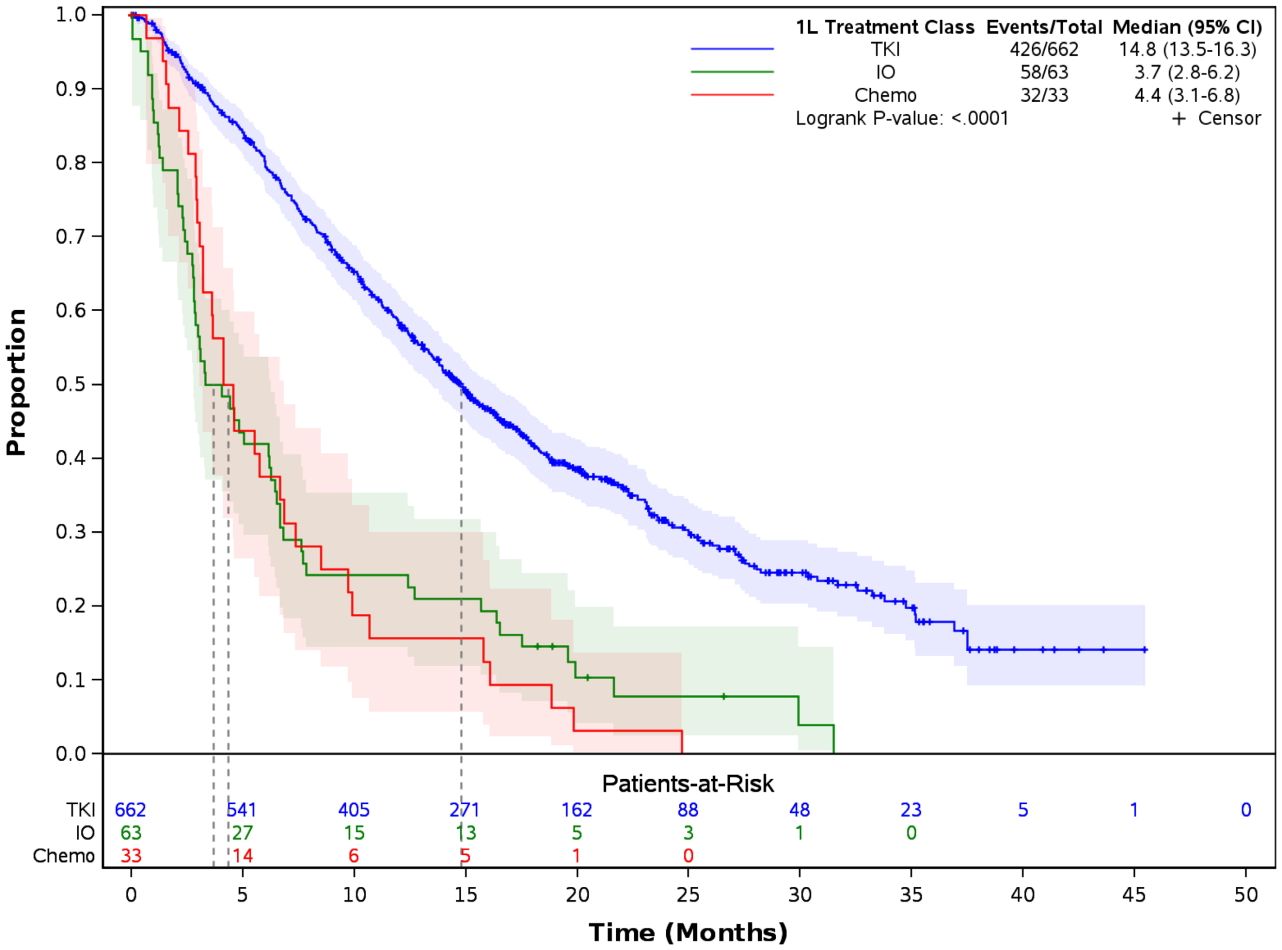
Time to next treatment or death among patients with stage IV EGFRm NSCLC^1^. 1L, first line; CI, confidence interval; EGFR, epidermal growth factor receptor, EGFRm, EGFR mutation; HR, hazard ratio; IO, immunotherapy; NSCLC, non‐small cell lung cancer, TKI; tyrosine kinase inhibitors. ^1^Patients with no evidence of a subsequent treatment were censored at the end of data availability (June 30, 2020).

Median TTNTD was longer for patients treated with EGFR TKI (Figure 2; 14.8 months, 95% CI: 13.5–16.3) compared to patients treated with IO (3.7 months, 95% CI: 2.8–6.2, *p* < 0.001) and chemotherapy (4.4 months, 95% CI: 3.1–6.8, *p* < 0.001). After adjustment with Cox regression, patients treated with EGFR TKI had significantly lower risk of initiating 2L therapy or death compared to patients on IO (HR: 0.33, 95% CI: 0.24–0.43, *p* < 0.001) or chemotherapy (HR: 0.34, 95% CI: 0.23–0.50, *p* < 0.001).

## 4 DISCUSSION

This retrospective study, based on EHR data from oncology practices in the United States, showed that physicians initiating IO or chemotherapy were more likely to initiate therapy before receiving guideline recommended testing. Specifically, in comparison to EGFR TKIs, physicians initiating 1L IO were nearly 20 times more likely to initiate before receiving EGFR testing results. Physicians initiating chemotherapy were almost 15 times more likely to initiate before receiving EGFR testing results in comparison to EGFR TKIs. As noted, approximately 9% of patients initiating EGFR TKIs, initiated before testing results were known. This may be due to discrepancies in when the prescribing physician was made aware of a positive test result and when it was officially documented in the EHR. In addition, adherence to NCCN Guidelines recommended EGFR TKI therapy was associated with better TTNTD in 1L EGFRm mNSCLC patients. Patients who received 1L EGFR TKI had significantly longer median TTNTD compared to patients who received IO or chemotherapy alone. The median TTNTD of 4.4 months for chemotherapy in this study is consistent with other real‐world studies,^18^ however, it is lower than PFS of EGFR patients treated with chemotherapy in clinical trials.^21^ There may be several reasons for this difference, notably, clinical trial populations tend to be younger, have a lower number of comorbid conditions, and better performance scores than real‐world populations. Another reason may be due to patients switching treatments after receiving test results. While patients that switched within 8 weeks were excluded, there could be uncommon instances where the testing results took longer than 8 weeks to report. Furthermore, higher acuity patients may be started on chemotherapy while awaiting test results. After baseline adjustments, the risk of 2L therapy initiation or death was significantly lower for the EGFR TKI therapy group versus IO or chemotherapy alone. This is expected as EGFR TKIs are tailored to specifically target proteins that have certain EGFRm, allowing for a more focused and effective treatment for these tumor cells compared to IO or chemotherapy.

In this study population, over 87% of patients received EGFR TKI in adherence to NCCN Guidelines recommendations, although around 8% received IO, and around 4% received chemotherapy alone as 1L therapy, consistent with a previous report.^12^ As expected, a higher proportion of patients receiving IO or chemotherapy initiated 1L therapy before EGFR test results were available. This highlights the need to wait for test results prior to making therapeutic decisions so that patients receive the most appropriate therapy and potentially improve their prognosis.

This study showed that during this time period, patients treated with 1L EGFR TKI had a 67% lower risk of subsequent therapy or death compared to patients treated with 1L IO among the EGFRm NSCLC population. The data support NCCN Guidelines that recommend treating this patient population with EGFR TKI, rather than IO, as 1L therapy. These results corroborate those reported in the real‐world studies examining TTNTD associated with 1L therapies among similar patient populations in the United States. In a study examining patients with stage IIIB/IV EGFRm NSCLC,^18^ Li et al. found significantly longer TTNTD among patients receiving EGFR TKI (13.1 months for erlotinib and 12.1 months for afatinib) compared to patients receiving non‐EGFR TKI targeted therapy (i.e., bevacizumab or cetuximab, 5.3 months), or chemotherapy (4.2 months). The study, however, did not include osimertinib patients, which had a later FDA approval date. Post‐hoc analysis by TKI generation in the current study (not shown) found that TTNTD for osimertinib patients was 18.7 months and TTNTD for first‐ and second‐generation TKIs was 8.7 and 12.6 months, respectively.

Timely biomarker testing is key for physicians to make treatment decisions. Reducing TAT for tissue biopsy is important so physicians can use test results in their decision making. Liquid biopsy can enable faster TAT of molecular test results.^22^ While initiating treatment as soon as possible may appear to be effective, this study showed better outcomes for patients that initiated appropriate therapy based on EGFR status. This reinforces consensus recommendations that suggest waiting for testing results before initiating 1L therapy.^23^

This study has several notable strengths. First, the Flatiron database, which includes both structured and unstructured data, is a nationally representative dataset that provides the unique opportunity to evaluate real‐world TTNTD in NSCLC patients. Second, the data are mainly drawn from community‐based oncology practices (89%) and therefore the results are generalizable to United States oncology patients treated in community practice settings (especially due to the demographic and geographic diversity of the Flatiron data), which account for approximately 80% of oncology services provided in the United States. Lastly, this is the first real‐world study to directly compare TTNTD between 1L EGFR TKI and IO in EGFRm NSCLC patients. It is important to show data in comparison to IO therapies as physicians tend to prescribe these therapies more than chemotherapy.

The study is not without limitations. First, baseline comorbidities and conditions that may have led to the exclusion of patients from the study sample were identified by diagnosis codes in the EHR, which are likely incomplete because the data are derived from community oncology practices rather than general practitioners. Therefore, misclassification due to miscoding of these conditions may have resulted in the inclusion of patients with exclusionary conditions (e.g., patients diagnosed with other primary tumors). However, misclassification is expected to be non‐differential with respect to treatment groups and outcomes. Second, certain variables related to disease characteristics (e.g., ECOG status and non‐cancer comorbidities), as well as data on medication prescribed outside of the oncology clinic practice, may have been under‐recorded in the dataset. Furthermore, due to data unavailability, this study could not evaluate PFS or duration of response. Instead, TTNTD was used as a proxy for PFS, although there are limitations to using this measure. Sometimes treatment discontinuation and initiation of subsequent treatment may be a result of factors not related to disease progression. Fourth, the number of patients treated with IO and chemotherapy were lower in comparison to the EGFR TKI group, however, this is expected due to the lack of guideline recommendations for these therapies in EGFRm NSCLC patients. Lastly, as with any retrospective observational study, there is the potential for residual confounding due to unmeasured or unobservable confounders, such as sites of metastasis and other prognostic variables.

## 5 CONCLUSIONS

The current study demonstrates the real‐world clinical benefit of EGFR TKIs over IO or chemotherapy, among patients with stage IV EGFRm NSCLC. This study highlights the importance of adhering to NCCN Guidelines and initiating EGFR TKIs as a 1L treatment among this patient population. Waiting to initiate 1L therapy until after testing results are received, when clinically feasible, may help ensure that providers are prescribing the appropriate treatment while minimizing impact of suboptimal regimens and associated side effects.

## AUTHOR CONTRIBUTIONS

**Jon Apple:** Investigation (equal); writing—original draft (lead). **Rahul Shenolikar:** Investigation (equal); writing—review and editing (equal). **Kevin De Silva:** Formal analysis (equal); writing—review and editing (equal). **Ping Sun:** Formal analysis (equal); writing—review and editing (equal). **Alexander Spira:** Investigation (equal); writing—review and editing (equal).

## ETHICS STATEMENT

The fully de‐identified data used for this analysis are not subject to ethics committee approval.

## Data Availability

Data are available from the authors with the permission of AstraZeneca. The data that support the findings of this study are available from the corresponding author, JA, upon reasonable request.
